# The Treatment of Diarrhœa by Oxide of Zinc

**Published:** 1878-11

**Authors:** 


The Treatment of Diarrhœa by Oxide of Zinc.—Dr. Jacquier has followed, in the service of Dr. Bonamy, at Nantes, the good effects of the employment of oxide of zinc in diarrhœa. The formula which he has employed is the following: Oxide of zinc, 54 grains: bicarbonate of soda, 7½ grains; in four packets, one to be taken every six hours. In all the cases which he observed, oxide of zinc produced rapid cure of diarrhœa. In fourteen cases observed by Puygautier, the cure was even more rapid, since in only one case were three doses of the medicine required. The results are considered to have been more satisfactory, inasmuch as in several cases the malady had endured from one to many months, and other methods of treatment had not produced any improvement. Thus he concludes that, although by no means to be held as exclusive treatment, the employment of oxide of zinc deserves to be more generally known as useful in diarrhœa.—*British Medical Journal*, Sept. 28, 1878.

